# A Fundamental Change in Antibiotic Susceptibility Testing Would Better Prevent Therapeutic Failure: From Individual to Population-Based Analysis

**DOI:** 10.3389/fmicb.2020.01820

**Published:** 2020-08-18

**Authors:** Ivan Brukner, Matthew Oughton

**Affiliations:** ^1^Department of Diagnostic Medicine, Jewish General Hospital, Montreal, QC, Canada; ^2^Molecular and Regenerative Medicine, Lady Davis Institute for Medical Research, Montreal, QC, Canada; ^3^Faculty of Medicine, McGill University, Montreal, QC, Canada

**Keywords:** antibiotic susceptibility, antibiotic resisitance, bacterial growth, qPCR, microbiology, guide to therapy, laboratory results, immunocompromidsed patients

Most patients with an acute bacterial infection have immune defense mechanisms to respond to the pathogen and destroy both resistant and non-resistant bacterial cells, facilitating the elimination of the pathogen. However, a growing population of patients–those with an immune deficiency—may lack some, or many aspects of those defense mechanisms, and thus require effective antibiotic therapy to survive or reduce their time required for recovery. Antibiotic Susceptibility Testing (AST) can help in choosing the most appropriate option. However, misguiding AST results can lead to poor and even fatal outcomes (Zilberberg et al., 2017; Jiang et al., 2018; Karve et al., 2018; Band and Weiss, 2019; Eliakim-Raz et al., 2019; Peeters et al., 2019; Rodriguez-Gomez et al., 2019; Uppsala University, 2019). There is a consensus among some medical researchers that these tests need improvement (Valsesia et al., 2015; Karve et al., 2018; Martin et al., 2018; Band and Weiss, 2019; Kahlmeter et al., 2019; Lerminiaux and Cameron, 2019; Nicoloff et al., 2019; Peeters et al., 2019). To improve the clinical value of AST, we challenge and propose a change of the two most dogmatic steps of the clinical tests: standardization of bacterial inoculation size (McFaland, 1907; Smith and Kirby, 2018) and the use of a limited number of bacterial colonies (Kao et al., 2014; Qin et al., 2018; Maciel et al., 2020; Montealegre et al., 2020). Problems with AST can be resolved by replacing the current approach that relies on selection of a small number of colonies with a population-level approach.

Two leading organizations that set standards for AST, the European Committee on Antimicrobial Susceptibility Testing (EUCAST) and the Clinical and Laboratory Standards Institute (CLSI), acknowledge that there are challenges associated with the interpretation of their recommended tests (Kahlmeter et al., 2019). They have used different strategies to deal with these challenges. One such strategy, implemented by EUCAST, was to coin the term “technical uncertainty.” Use of this term was intended to attenuate small, uncontrolled technical factors from causing significant discrepancies in interpretations (Kahlmeter et al., 2019). On the contrary, CLSI acknowledged the inherent uncertainty of the test, without insisting it was solely due to technical factors (Kahlmeter et al., 2019). Future changes in regulation and standardization of AST would need to be convergent and to facilitate clinical interpretation of the results.

The response to antibiotics within bacterial populations is inherently variable due to the unknown genetic complexity of the population, as previously suggested by some authors (Qin et al., 2018; Kahlmeter et al., 2019; Mouton et al., 2019; Nicoloff et al., 2019). Present formats of AST are designed to detect the susceptibility of the most prevalent bacteria, but not necessarily the most resistant. Other bacterial subtypes with higher resistance than the majority of the bacterial population may be present at lower frequencies in clinical samples, but remain unanalyzed (Martin et al., 2018; Qin et al., 2018; Maciel et al., 2020; Montealegre et al., 2020). Unfortunately, diversity in susceptibility remains undetected due to the current standard operating procedures of AST. As a common example Figure 1 demonstrates typical but contradictory logical reasoning between antibiotic screening results and AST, which can be reproduced in any clinical microbiology laboratory. What is the cumulative probability (Pc) of getting “resistant result” from hetero-resistant samples with different relative frequencies of resistant (Rf), vs. sensitive bacterial cells present in the sample? Cumulative binomial probability (probability of getting a result indicating resistance, or Pc, in Figure 1A), refers to the probability that the value of a binomial random variable falls within a specified range. The table from Figure 1A demonstrates that even with a Rf of 0.25, the chances of detecting resistance by selecting only 5 colonies/cells is only 0.76. If the resistant cells are 10x less frequent (Rf = 0.025), the probability drops to 0.12, with further decrease as the frequency of resistant cells continues to drop. However, by the application of a plate screening method, (see below), where 10,000–100,000 cells can be inoculated and screened on the same plate, the cumulative probability of getting resistant cells is quite good, even if relative frequencies are only 0.00025 of resistant vs. sensitive cells. Ultimately, a combination of species-specific bacterial qPCR detection combined with a brief incubation in the presence of antibiotic permits the detection of even rare resistant bacterial cells. A common clinical example is the urine culture (see Figure 1B), declared as positive for *E. coli* (more than 10^6^ colony forming units per mL of urine). AST, when performed routinely, determines the *E.coli* to be sensitive on ciprofloxacin. However, when the same sample is inoculated directly on agar containing 10 microgram/mL of ciprofloxacin, (MacConkey Agar with Ciprofloxacin, Hardy Diagnostics, VWR, Canada), resistance colonies were clearly detected (Figure 1B). These examples are indicating that AST methodology may yield inaccurate results for pathogens exhibiting hetero resistance.

**Figure 1:**
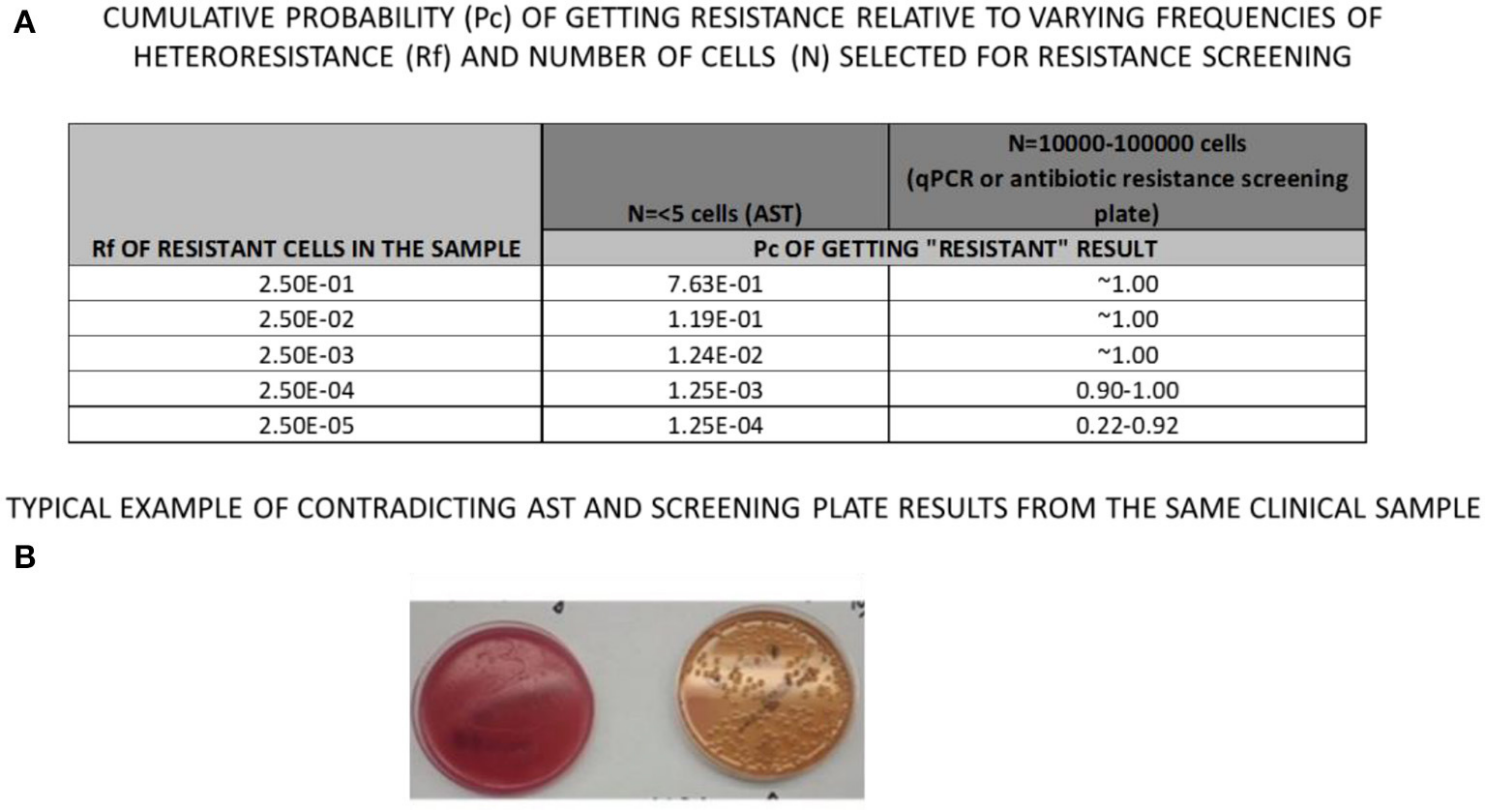
Same clinical sample was loaded on **(A)** blood Agar plate (left) forming confluent culture (more then 10,000,000 cells per ml) and on **(B)** MacConkey, Cipro-screening plate (right), revealing 200 resistant Colony Forming Units, CFU), contrary to Antibiotic Susceptibility test which reported only CIPRO-sensitive culture.

To predict response to antibiotic therapy, these undetected distribution-outliers—characterized by higher antibiotic resistance—should be considered.

As a reminder of standard operating procedures of AST in clinical microbiology, the first step of testing is to culture bacteria from the original sample on primary inoculum plates. The second step is to select a few individual colonies from the primary plate, prepare a standardized inoculum for AST (Kirby-Bauer plate), and determine the susceptibility result after overnight (16–18 h) incubation. Contrary to this approach, inoculating the original sample directly on antibiotic screening agar plate (Figure 1B) reveals antimicrobial resistance present in some bacteria from the inoculum. Therefore, antibiotic-resistant bacteria that were not detectable by conventional AST, were present at very low frequencies (≤1 in 10,000 colonies). This discrepancy is rooted in inoculum standardization and reduction of sample diversity when selecting individual colonies during these measurements.

There is a consensus among researchers that inoculum size has a dramatic impact on the test result. Standard tests use an inoculum size of 0.5 McFarland units (a measure of turbidity, ~10^8^ CFU/mL) (McFaland, 1907). The rationale for this decision was to eliminate variability due to inoculum size. However, bacterial abundance is inherently variable in each clinical sample. Furthermore, the higher the bacterial inoculum concentration is, the more likely the bacterial population will develop resistance. To illustrate, think of two hypothetical patients with the similar infections (same bacterial species). The first patient, X, has 100 times the burden of infection (bacteria per volume or mass of biological fluid/tissue) compared to the second patient, Y. With the current AST, samples from patients X and Y tested multiple times would, over time, produce the same susceptibility result. However, patient X's infection would have a higher propensity for developing resistance than patient Y's. Moreover, patient X's treatment would be more likely to fail due to this difference. Therefore, standardizing inoculum size will cause a loss of clinically valuable information if we are aiming to develop a new generation of AST.

The major limitation with current AST design is the need to select only a few isolated colonies. This practice limits the test to a small number of isolates from an often-complex bacterial population. Only progenitors of a few individual bacterial cells will be standardized to produce an inoculum size of 0.5 McFarland units. This critical step in sample processing typically reduces bacterial diversity by up to 10^6^ and leaves the result of the test to “pure” sampling chance, where statistics are not in favor of detecting resistance. Altogether, standardization of sample size and reduction of sample diversity both reduce the clinical value and predictive power of AST.

The predictive power of AST can improve, though, if bacterial species are analyzed in the context of the original clinical sample, reflecting the patient-specific burden of infection. A new generation of nucleic acid amplification tests (NAATs) can measure species-specific growth rates of bacteria in the original sample in response to different antibiotics (Rolain et al., 2004; Maxson et al., 2018). These tests require ~2–4 h of *in vitro* incubation, which would be a significant improvement in turnaround time (Luo et al., 2018). Furthermore, the current operative cost of these PCR-based tests can be below equivalent cost related to traditional plate-based tests.

In conclusion, the current evidence suggests that the present methodology of AST should be reconsidered. We must move forward, taking advantage of the new technology available, and abandon inoculum size standardization and the use of pure cultures. This might change the rules both for industry and the public sector but will ultimately benefit patients in need of antibiotic treatment, especially those that are immunocompromised.

## Author Contributions

All authors listed have made a substantial, direct and intellectual contribution to the work, and approved it for publication.

## Conflict of Interest

The authors declare that the research was conducted in the absence of any commercial or financial relationships that could be construed as a potential conflict of interest.
